# Rhizoengineering with biofilm producing rhizobacteria ameliorates oxidative stress and enhances bioactive compounds in tomato under nitrogen-deficient field conditions

**DOI:** 10.1016/j.heliyon.2024.e34276

**Published:** 2024-07-08

**Authors:** Md. Manjurul Haque, Md. Rahat Bari Rupok, Abul Hossain Molla, Md. Mizanur Rahman, Habibul Bari Shozib, Md Khaled Mosharaf

**Affiliations:** a Department of Environmental Science, Faculty of Agriculture, Bangabandhu Sheikh Mujibur Rahman Agricultural University (BSMRAU), Gazipur, 1706, Bangladesh; b Department of Soil Science, Faculty of Agriculture, Bangabandhu Sheikh Mujibur Rahman Agricultural University (BSMRAU), Gazipur, 1706, Bangladesh; c Grain Quality and Nutrition Division, Bangladesh Rice Research Institute, Gazipur, Bangladesh; d Division of Agriculture and Environmental Science, School of Biosciences, University of Nottingham, Sutton Bonington, LE12 5RD, United Kingdom

**Keywords:** Rhizoengineering, Biofilm, Plant growth promoting rhizobacteria, Oxidative stress, Bioactive compounds, Tomato

## Abstract

Nitrogen (N) deficiency limits crop productivity. In this study, rhizoengineering with biofilm producing rhizobacteria (BPR) contributing to productivity, physiology, and bioactive contents in tomato was examined under N-deficient field conditions. Here, different BPR including *Leclercia adecarboxylata* ESK12, *Enterobacter ludwigii* ESK17, *Glutamicibacter arilaitensis* ESM4, *E. cloacae* ESM12, *Bacillus subtilis* ESM14, *Pseudomonas putida* ESM17 and *Exiguobacterium acetylicum* ESM24 were used for the rhizoengineering of tomato plants. Rhizoengineered plants showed significant increase in growth attributes (15.73%–150.13 %) compared to the control plants. However, production of hydrogen peroxide (21.49–59.38 %), electrolyte leakage (19.5–38.07 %) and malondialdehyde accumulation (36.27–46.31 %) were increased remarkably more in the control plants than the rhizoengineered plants, thus N deficiency induced the oxidative stress. Compared to the control, photosynthetic rate, leaf temperature, stomatal conductance, intrinsic and instantaneous water use efficiency, relative water content, proline and catalase activity were incredibly enhanced in the rhizoengineered plants, suggesting both non-enzymatic and enzymatic antioxidant systems might protect tomato plants from oxidative stress under N-deficient field conditions. Yield (10.24–66.21 %), lycopene (4.8–7.94 times), flavonoids (52.32–110.46 %), phenolics (9.79–23.5 %), antioxidant activity (34.09–86.36 %) and certain minerals were significantly increased in the tomatoes from rhizoengineered plants. The principal component analysis (PCA) revealed that tomato plants treated with BPR induced distinct profiles compared to the control. Among all the applied BPR strains, ESM4 and ESM14 performed better in terms of biomass production, while ESK12 and ESK17 showed better results for reducing oxidative stress and increasing bioactive compounds in tomato, respectively. Thus, rhizoengineering with BPR can be utilized to mitigate the oxidative damage and increase the productivity and bioactive compounds in tomato under N-deficient field conditions.

## Highlights

• Rhizoengineereing enhances productivity of tomato in N-deficient field conditions.
• Yield and bioactive compounds in tomato increase by rhizoengineering.
• Biofilm-mediated rhizoengineered plants ameliorate oxidative stress.
• PCA results confirm increased plant performance with rhizoengineering.

## 1 Introduction

Increasing crop yield to fulfill the demand of growing world population is one of the major challenges in 21st century [1]. To achieve the target, farmers rely heavily on the extensive application of chemical fertilizers and pesticide in their fields [2]. On average, about 50 % of yield increase has been reported with the application of chemical fertilizers in crop production [3]. But the negative impact of these intensive practices in agriculture is now becoming much more visible as crop lands are losing fertility [4]. Climate change is also playing a crucial role and making the situation much worse [5]. Hence getting the expected yield from crop lands is becoming tougher day by day.

Nitrogen (N), phosphorus (P) and potassium (K) are the key plant macronutrients which limit crop productivity massively. Among macronutrients, N is the vital component of nucleotides, proteins, enzymes, ATP, cAMP, phosphoinostides, and chlorophylls [[6], [7], [8], [9], [10], [11]]. N is required for enzyme production, nutrient uptake, water balance, signaling pathways, photosynthesis and respiration rate in plants, thus ultimately influencing growth, development and yield [9,[12], [13], [14], [15]]. N-deficient plants also induce oxidative stress by generating the harmful reactive oxygen species (ROS) including superoxide anion (O_2_^−^), hydrogen peroxide (H_2_O_2_), and hydroxyl radicals (OH^•^) [16]. ROS causes damage to lipids, proteins, and DNA [17]. To counteract the adverse effect of ROS, N-deficient plants synthesize non-enzymatic antioxidants, such as phenylpropanoids, starch, flavonoids, anthocyanin and pigments e.g., chlorophylls and carotenoids [[18], [19], [20], [21], [22]]. Nevertheless, N-deficient plants also generate enzymatic antioxidants like superoxide dismutase (SOD), peroxidases (POD), catalases (CAT), ascorbate peroxidase (APX) and glutathione reductase (GR) to detoxify ROS [16,23]. So, supplying sufficient N to the soil is an effective way of maintaining proper plant health.

The most common way of supplying N to the soil is by application of synthetic N fertilizer. Supplying N to crops is a major challenge as it offers only 20–30 % of use efficiency in cereals [24]. According to the IPCC (Intergovernmental Panel on Climate Change) report, since the 1960s, the application of synthetic N fertilizers has increased by 800 % [25]. So, most of N is wasted and contributes to environmental pollution by leaching, runoff, volatilization, and releasing N_2_O, a potent greenhouse gas reported to destroy ozone, ultimately contributing to global warming [26]. Therefore, sustainable eco-friendly alternatives to synthetic chemicals are urgently required to address the forthcoming challenges.

Rhizoengineering (RE) with plant growth-promoting rhizobacteria could replace synthetic fertilizers, enhancing crop quality as well as soil health [27]. The rhizosphere, near plant roots, serves as the hotspot for diverse microbes which are influenced by plant-released compounds like carbohydrates, organic acids, and amino acids [27]. Manipulating this microbiome through rhizoengineering can boost plant growth and address agricultural challenges like carbon sequestration [28] and greenhouse gas reduction [29]. Although managing the entire microbiome is complex, the most direct and eco-friendly method is introducing artificially cultured microbes through inoculation [30].

The rhizosphere microbiome is primarily comprised of plant growth-promoting rhizobacteria (PGPR), with only a small portion being plant pathogens [31]. PGPR promote plant growth through various mechanisms, including providing nutrients (nitrogen fixation, mineral nutrient solubilization), producing phytohormones, secreting volatile compounds, producing hydrolytic enzymes, inducing systemic resistance against pests and pathogens, mitigating abiotic stresses, and biocontrol of phytopathogens [[32], [33], [34], [35], [36], [37], [38], [39]]. Genera of reported PGPR include *Acinetobacter*, *Agrobacterium*, *Azospirillum*, *Pseudomonas*, *Bacillus*, *Rhizobium*, *Enterobacter*, *Pantoea* and others [[40], [41], [42], [43], [44], [45], [46]]. PGPR not only increase plant growth but also improve fruit quality and alleviate various stresses such as drought, salinity, heat, cold and heavy metals [[47], [48], [49]]. However, some studies indicate that PGPR strains may perform better in greenhouse or laboratory conditions than in field conditions [32].

Bacterial biofilms are defined as an underlying aggregation of bacteria protected in self-encased extracellular polymeric substance (EPS) which attach to any surfaces either living or non-living [50]. EPS comprises of different biomolecules i.e., proteins, lipids, polysaccharides, extracellular nucleic acids, aminopeptidase, and bacterial cellular appendages e.g., flagella, curli fimbriae, and type IV pili [[51], [52], [53]]. Biofilm producing rhizobacteria (BPR) enhance the survivability because the EPS around the cell provides extra protection against antibiotics, xenobiotic compounds like toxic heavy metals, and dyes, and abiotic stressors including salinity, drought, and heat, thus improving survival rates under field conditions compared to their planktonic counterparts [[54], [55], [56], [57]]. So, rhizoengineering with BPR could be of great advantage.

Tomato (*Solanum lycopersicum* L.) is well known for its high nutritional value which not only includes proteins, lipids, and sugar but also antioxidants e.g., carotenoids (β-carotene and lycopene), phenolics, ascorbic acids and flavonoids, that are essential for human health [58,59]. Almost every country in the world consumes tomatoes on a daily basis, both fresh and processed in forms such as paste, soup, juice, powder and concentrates [60]. Some PGPR strains i.e., *Bacillus subtilis*, *B. amyloliquefaciens, B. licheniformis*, *Kosakonia Radicincitans* and *Priestia megaterium* has been reported to enhance yield, total soluble solids (TSS) and lycopene content of tomato [[61], [62], [63]]. A few biofilm producing PGPR (BPPGPR) strains isolated from heat-prone ecosystem of Bangladesh has been found to increase yield, TSS, lycopene, total flavonoids, phenolics and total antioxidant capacity in pot-grown tomatoes [54]. Recently, several BPPGPR strains has been isolated from the rhizosphere of tomato plants grown in the saline affected areas of Bangladesh whose PGPR properties been tested in the laboratories [64]. The plant growth promoting properties of these identified bacterial strains include nitrogen (N) fixation, nutrient solubilization [e.g., P, K, iron (Fe) and zinc (Zn)], indole-3-acetic acid (IAA) production, production of 1-aminocyclopropane-1-carboxylate (ACC) deaminase, ammonia (NH_3_), hydrogen cyanide (HCN), EPS, and bioflm [64]. However, rhizoengineering with these promising BPPGPR strains contributing to productivity, mitigation of oxidative stress, and production of bioactive compounds in tomato under N-deficient field conditions is yet to be studied.

So, the objective of this study was to assess the effects of rhizoengineering with specific biofilm-forming PGPR strain on growth, physiology, yield, as well as to quantify the production of antioxidant compounds in tomato under N-deficient open field conditions.

## 2 Materials and methods

### 2.1 Experimental field

A field experiment of tomato was conducted during winter season (October to April). The field is situated at 24.09°N latitude and 90.26°E longitude. According to USDA soil classification, soil of this site is Salna silty clay loam in the Madhupur tract agro-ecological zone (AEZ) of Bangladesh under the order of Inceptisols. The area is high land and characterized by subtropical climatic conditions with an average summer temperature of 29 °C and winter temperature 18 °C. The soil attributes of the experimental field include organic matter = 1.16 %, pH = 6.49, total N = 0.09 %, available P = 7.75 mg kg^−1^ and exchangeable K = 140.4 mg kg^−1^, suggesting that this field was low fertile specifically N-deficient.

### 2.2 Raising seedlings and land preparation

BARI Tomato 8, a high yielding tomato variety, was used as the test crop. The seeds of this variety were procured from Vegetable section of Horticulture Center at Bangladesh Agricultural Research Institute (BARI), Gazipur, Bangladesh. Initially, 5 % NaOCl sterilized seeds were sown in a raised seedbed. The land was prepared properly and made into three small blocks (24.0 m × 1.5 m). For achieving the high-yield goal, the recommended N:P:K dose were estimated from the model provided by Bangladesh Agricultural Research Council [65]. 1/3rd of the recommended N:P:K dose were applied during the final field preparation as basal dose in every block. Other than this, no fertilization was done during the experimental period.

### 2.3 Experimental design and treatments

In order to evaluate how rhizoengineering with BPPGPR strains impact tomato productivity, a field experiment was conducted using a randomized complete block design (RCBD) having three sets of repetitions. A total of eight (08) treatments including non-inoculated control and seven BPPGPR strains previously isolated and identified from the saline prone ecosystem of Bangladesh having plant growth-promoting (PGP) properties [64] i.e., *Leclercia adecarboxylata* ESK12, *Enterobacter ludwigii* ESK17, *Glutamicibacter arilaitensis* ESM4, *E. cloacae* ESM12, *Bacillus subtilis* ESM14, *Pseudomonas putida* ESM17 and *Exiguobacterium acetylicum* ESM24 (available on NCBI database) were used in this experiment. Each plot size was maintained at an area of 2.5 × 1.0 m^2^.

### 2.4 Rhizoengineering and transplanting of seedlings

Selected strains were cultured in 250 mL yeast extract peptone broth at 28 °C in shaking conditions for 12 h. Afterwards, the cells of each culture were collected by centrifugation. The pellets were re-suspended in deionized water and diluted to ca. 10^9^ CFU mL^−1^. Evenly-sized, healthy 22-day-old tomato seedlings were carefully pulled up from the seedbed without damaging the roots. In the case of rhizoengineering, treatment-wise roots of tomato seedlings were submerged in bacterial suspension. For control or non-inoculated treatment, the seedlings’ roots were submerged in deionized water. After 2 h of incubation, a single seedling was transplanted in each pit. The spacing maintained in each plot was row to row 0.5 m and plant to plant 0.5 m. By maintaining this spacing, 10 plants were planted in each plot. A 10 mL additional bacterial culture of each treatment (grown and harvested as described earlier) was diluted in deionized water (ca. 10^9^ CFU mL^−1^) and applied to the soil around the plant base (rootzone) of each plant on 30- and 60-days post planting (DPP). For control, only deionized water was applied at the plant base of each plant. Standard management practices i.e., irrigation, weeding, mulching and staking were followed but no herbicides or pesticides were applied as there was no pathogen or pest attack observed in the field.

### 2.5 Vegetative growth, dry matter, yield and biochemical parameters

Three plants from each plot were randomly selected for data collection. For achieving high precision plant selection was done in a manner that the border effect was avoided. At 50 % flowering stage, parameters i.e., height, leaf number, primary branches, maximum leaf length and width, and biomass accumulation were recorded. For dry matter weights, the shoot and root of individual plant were separately oven dried at 70 °C. At 50 % flowering stage, the leaves were also collected for the biochemical parameters. For yield, fully ripe tomatoes were hand-picked at 3–5 day intervals, weighed, and total yield was stated as kg plant^−1^.

### 2.6 Relative water content (RWC)

Five round shaped leaf discs (1 cm^2^) were collected from fully developed 3rd leaf from top of each selected plant and weighed. The collected leaf discs were then submerged in deionized water at 4 °C for 24 h and the turgid weight was recorded afterwards. Then discs were dried in an oven at 72 °C and weight was noted. The following formulae was used to calculate RWC:

$$\text{RWC}\;\left(\%\right)=\left[\;\frac{\text{Fresh}\;\text{wt}\;\text{of}\;\text{disc}\;‐\;\text{Dry}\;\text{wt}\;\text{of}\;\text{disc}}{\text{Turgid}\;\text{wt}\;\text{of}\;\text{disc}\;‐\;\text{Dry}\;\text{wt}\;\text{of}\;\text{disc}}\;\right]\times 100$$

### 2.7 Gas exchange features

Photosynthetic parameters such as photosynthetic rate (Pn), leaf temperature (LT), stomatal conductance to H_2_O (g_s_) and transpiration rate (E) were recorded with a portable photosynthesis system (Model: LI-6400XT System, LI-COR Biosciences, Lincoln, Nebraska, USA) under bright sunlight. Using the obtained data for the abovementioned parameters, instantaneous water use efficiency (WUE_ins_) and intrinsic water use efficiency (WUE_int_) were determined by applying the following formula:

$${\text{WUE}}_{\text{ins}}\;\left(\mu M\;{\text{CO}}_{2}{\;M}^{‐1}{\;H}_{2}O\right)=\frac{\text{Photosynthetic}\;\text{rate}\;\left(\text{Pn}\right)}{\text{Transpiration}\;\text{rate}\;\left(E\right)}$$

$${\text{WUE}}_{\mathrm{int}}\;\left(\mu M\;{\text{CO}}_{2}{\;M}^{‐1}{\;H}_{2}O\right)=\frac{\text{Photosynthetic}\;\text{rate}\;\left(\text{Pn}\right)}{{\text{Stomatal}\;\text{conductance}\;\text{to}\;H}_{2}O\;\left(g_{s}\right)}$$

### 2.8 Chlorophylls and carotenoids

The chlorophyll (Chl) pigments i.e., Chl *a*, Chl *b*, total Chl and carotenoids of tomato leaf samples were measured as described by Khan et al. [58] using UV–vis spectrophotometer. The following formulas were used for calculating chlorophylls and carotenoids.

$$\text{Chl}\;a=\left[12.7\left(A663\right)\;‐\;2.69\left(A645\right)\right]\left[\frac{V}{1000\times W}\right]$$

$$\text{Chl}\;b=\left[22.9\left(A645\right)\;‐\;4.68\left(A663\right)\right]\left[\frac{V}{1000\times W}\right]$$

$$\text{Total}\;\text{Chl}=\left[20.21\left(A645\right)\;+\;8.02\left(A663\right)\right]\left[\frac{V}{1000\times W}\right]$$

$$\text{Carotenoids}=\frac{\left[1000\left(A470\right)\;‐\;2.270\times \text{Chl}\;a\;‐\;81.4\times \text{Chl}\;b\right]}{227}$$

Here, A (663, 645, 470) denotes the absorbance of the extracted chlorophyll pigment at 663, 645 and 470 nm wavelength, V indicates the acetone volume (mL) and W is the fresh leaf sample weight (g). The units were expressed in mg g^−1^ leaf fresh weight (FW).

### 2.9 Determination of electrolyte leakage, lipid peroxidation and hydrogen peroxide level

The method for assessing electrolyte leakage (EL) in the leaves was adapted from Lutts et al. [66] with minor modifications. Initially, five round leaf discs, each measuring 1 cm^2^, were placed in test tubes containing 10 mL of distilled water, and the electrical conductivity (EC) was recorded. Subsequently, the test tubes were placed in a water bath at 40 °C for 30 min, and the EC was measured again. Then, the tubes were heated to 100 °C, and after an additional 30 min, the EC was recorded once more. The following formula was utilized to calculate the EL.

$$\text{EL}\left(\%\right)=\left[\frac{\text{EC}\;\text{at}\;40\;{}^{\circ}C-\text{EC}\;\text{at}\;\text{room}\;\text{temperature}}{\text{EC}\;\text{at}\;100\;{}^{\circ}C}\right]\times 100$$

Lipid peroxidation in relation to malondialdehyde (MDA) content in tomato leaves were measured as described in Vemanna et al. [67]. The quantification of hydrogen peroxide (H_2_O_2_) levels in tomato leaves was done following the procedure outlined by Yu et al. [68].

### 2.10 Determination of proline, catalase and ascorbate peroxidase activity

The proline content in tomato leaves was determined by following the method of Bates et al. [69]. The catalase and ascorbate peroxidase (APX) activity in tomato leaves were assessed in accordance with the method described in Haque et al. [70] and Nakano and Asada [71], respectively.

### 2.11 Estimation of total antioxidant, total phenolic and flavonoids content

The antioxidant potential of fully ripe tomato fruits was evaluated using the 1,1-diphenyl-2-picryl hydrazyl (DPPH) radical scavenging assay, as outlined in the methodology of Mensor et al. [72]. Total phenolic content of ripened tomato fruits were measured spectrophotometrically using the Folin–Ciocalteu method described in Ainsworth and Gillespie [73]. The obtained values were then compared with a standard curve of gallic acid and stated as per gram fruit weight gallic acid equivalents (μg g^−1^ FW GAE). Flavonoid content in tomato fruits were measured following the method in Zhishen et al. [74]. A standard curve of catechin was used to calculate the flavonoid content and expressed as per gram fruit weight catechin equivalent (μg g^−1^ FW CE).

### 2.12 Estimation of β-carotene and lycopene

β-carotene and lycopene were determined as described in Nagata and Yamashita [75]. In brief 1g ripe tomato was taken and macerated with mortar and pestle. Then mixed with 10 mL of hexane:acetone solution with a ratio of 4:6 and filtered through filter paper (Whatmann No. 42). The absorbance of the filtered sample was recorded at 663, 645, 505 and 453 nm consecutively with a spectrophotometer. The following formulas were used for calculating β-carotene and lycopene.

$${\beta ‐\text{carotene}=0.216\;A}_{663}{\;‐\;1.22\;A}_{645}{\;‐\;0.304\;A}_{505}+0.452\;A_{453}$$

$${\text{Lycopene}=‐\;0.0458\;A}_{663}{\;+\;0.204\;A}_{645}\;{+\;0.372\;A}_{505\;}‐\;0.0806\;A_{453}$$

### 2.13 Estimation of total soluble solid and total carotenoid content

Total soluble solids (TSS) were measured immediately after harvesting ripe fruits using a handheld refractometer. The determination of total carotenoid content (TCC) was conducted following the procedure outlined in Lachman et al. [76]. TCC in the fruit was compared to a standard lutein curve and expressed as lutein equivalent (LE) in micrograms per gram of fresh weight (μg g^−1^ FW LE).

### 2.14 Determination of mineral contents in tomato fruits

A Shimadzu ICP-AES (model ICP9820, Japan) was employed to measure the minerals (Na, K, Ca, Mg, Fe, Cu, and Zn) in ripe tomato fruits. Initially, dried fruit samples underwent microwave digestion using an Ethos Easy instrument from Milestone (USA), following the recommended 14-vessel program. Solutions for calibration studies were prepared by appropriately diluting ICP multi-element standard solution, with concentrations of 50 mg L^−1^ for K and 10 mg L^−1^ for Na, Ca, Mg, Fe, Cu, and Zn. These standard solutions were obtained from Sigma-Aldrich (TraceCERT, CRM 90243). Sample pre-treatment and dilution were carried out using deionized water with a maximum resistivity of 18.2 MΩ cm^−1^, obtained from a Direct-Q UV water purification system, and Suprapure 69 % HNO_3_ (m/m, Sigma Aldrich, USA).

### 2.15 Statistical analysis

All the experiments in this study except the field experiment, followed a completely randomized design (CRD) and were conducted with a minimum of three replications, each repeated at least twice. To assess various parameters in response to bacterial treatments, one-way ANOVA test was utilized. To confirm the normal distribution of the data sets, we conducted the Shapiro-Wilk test. Furthermore, Bartlett's test was performed to assess the homoscedasticity of the data, and it indicated that the data sets were indeed homogenous. The analysis, including the ANOVA test, examination of data distribution, assessment of variance homogeneity, and mean comparisons, was carried out using R Studio software. F and P values obtained from ANOVA test for all the parameters examined in this study are presented in supplementary table (Supplementary Table 1). For mean comparison, Fisher's Least Significant Difference (LSD) test was used. Additionally, R Studio was employed to generate Principal Component Analysis (PCA) and clustered heat maps based on the normalized data of different variables in respect to the treatments.

## 3 Results

### 3.1 Rhizoengineering with BPPGPR-strains improves growth-promoting traits in tomato

Tomato plants were grown in N-deficient field conditions in order to study the effect of rhizoengineering with *L. adecarboxylata* ESK12, *E. ludwigii* ESK17, *G. arilaitensis* ESM4, *E. cloacae* ESM12, *B. subtilis* ESM14, *P. putida* ESM17 or *E. acetylicum* ESM24 on growth in terms of plant height, leaf number, number of primary branches, maximum leaf length and width, and dry matter accumulation. All of these traits were recorded at 50 % flowering stage. All the examined traits were significantly (P < 0.05) influenced by the rhizoengineering with BPPGPR strains (Table 1). Plant height was increased by 16.27–28.96 % after bacterial application compared to the control plants (Table 1). The tallest plant (101.23 cm plant^−1^) was found by application of ESM17. However, plant height was statistically alike in ESM17, ESK12, ESM4, ESM12, ESM14 and ESM24 treatments. The shortest plant (78.5 cm plant^−1^) was noted in the control treatment. A significant improvement of the number of leaves (15.73–74.32 % increase over control) was also observed by rhizoengineering with BPPGPR strains (Table 1). The ESK12-inoculated plants produced the highest number of leaves (40.67 plant^−1^). The lowest number of leaves (23.33 plant^−1^) was found in the control treatment which was not considerably varied with ESK17 and ESM17. The number of primary branches in both treated and non-treated (control) plants were statistically non-significant (data not shown). A significant difference was recorded in the case of maximum leaf length and width by application of bacterial strains. ESM4 and ESM14-inoculated plants increased the leaf length by 23.32 and 24.97 %, respectively in relation to control plants (Table 1). The highest leaf width (32.23 cm) was also found by application of ESM24 (Table 1). Except ESM17, all the bacterized treatments produced 6.71–61.63 % more root dry biomass over the control treatment (Table 1). The maximum root dry biomass (3.37 g plant^−1^) was found by rhizoengineering with ESM4, followed by ESM14 (3.14 g plant-1). The minimum root dry biomass (2.02 g plant^−1^) was recorded in ESM17-treated plants which was statistically akin to control treatment, ESK12 and ESM24. In terms of shoot dry biomass production, all the rhizoengineered plants produced significantly higher values, ranging from 49.16 to 150.10 % increment over the control (Table 1). The highest shoot dry biomass (37.09 g plant^−1^) was obtained by rhizoengineering with ESM4 which differed significantly from all other treatments including control followed by ESM14-treated plants (32.33 g plant^−1^). The lowest shoot dry biomass (14.83 g plant^−1^) was recorded in control treatment.

**Table 1:** BPPGPR strains influenced on tomato plant growth parameters.

Treatments	Plant height (cm)	Number of leaves per plant	Maximum leaf length (cm)	Maximum leaf width (cm)	Dry matter production (g per plant)	
					Root	Shoot
Control	78.5 ± 2.33 c	23.33 ± 1.33 e	30.27 ± 1.71 d	22.00 ± 2.18 d	2.09 ± 0.21 d	14.83 ± 0.43 f
ESK12	97 ± 4.06 ab	40.67 ± 0.88 a	33.73 ± 1.43 bcd	28.30 ± 2.50 abc	2.46 ± 0.15 cd	27.42 ± 0.70 d
ESK17	91.27 ± 2.87 b	27.00 ± 1.15 de	31.83 ± 0.33 cd	27.53 ± 2.30 abc	2.72 ± 0.21 bc	28.37 ± 0.12 cd
ESM4	97.77 ± 2.77 ab	34.67 ± 2.60 b	37.33 ± 1.22 ab	29.60 ± 1.31 ab	3.37 ± 0.24 a	37.09 ± 0.41 a
ESM12	98.5 ± 2.86 ab	32.67 ± 2.85 bcd	35.00 ± 1.04 abc	26.70 ± 0.68 bcd	2.66 ± 0.01 bc	29.93 ± 1.21 c
ESM14	95.27 ± 3.34 ab	29.67 ± 2.03 bcd	37.83 ± 1.09 a	28.17 ± 0.44 abc	3.14 ± 0.13 ab	32.33 ± 0.93 b
ESM17	101.23 ± 3.60 a	27.67 ± 1.86 cde	32.33 ± 1.48 cd	24.17 ± 0.44 cd	2.02 ± 0.20 d	22.12 ± 1.01 e
ESM24	98.47 ± 3.03 ab	33.00 ± 1.53 bc	32.33 ± 0.93 cd	32.23 ± 2.31 a	2.23 ± 0.18 cd	23.74 ± 0.50 e

Data represents means ± standard errors obtained from three replicated treatments. Different letters in column indicate significant difference among treatments as determined by Fisher's least significant difference (LSD) test at 5 % (P < 0.05) level.

### 3.2 Rhizoengineering with BPPGPR-strains reduced oxidative damage in tomato plants

**Fig. 1:**
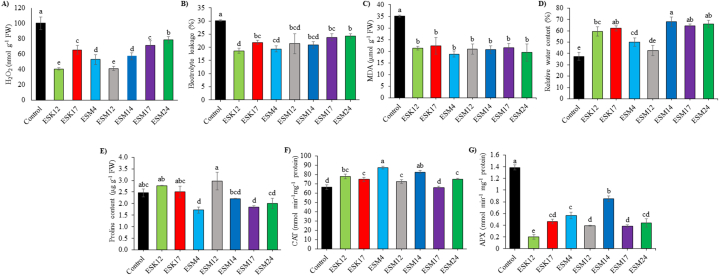
Role of BPPGPR strains on antioxidants and stress related parameters on tomato leaves. Hydrogen peroxide (H_2_O_2_) (A), electrolyte leakage (EL) (B), MDA (C), relative water content (D), proline content (E), CAT (F) and APX (G). The distinct letters above the vertical bars signify statistically significant differences among treatments at a significance level of 5 % (P < 0.05), as determined by Fisher's LSD test. Additionally, the error bars are the standard error obtained from three replicated treatments.

The role of bacterial strains against oxidative stress was assessed by determining the hydrogen peroxide (H_2_O_2_) level, electrolyte leakage (EL), malondialdehyde (MDA) and the level of osmoprotectants and reactive oxygen species (ROS) detoxifying enzymes such as proline, catalase (CAT), and ascorbate peroxidase (APX), and relative water content (RWC) in tomato leaves (Fig. 1A–G). Rhizoengineering with BPPGPR strains remarkably reduced the H_2_O_2_ production by 21.49–59.38 % (Fig. 1A), electrolyte leakage by 19.50–38.07 % (Fig. 1B) and MDA by 36.27–46.31 % compared to the control treatment (Fig. 1C). Conversely, RWC, and proline were increased by 13.80–81.51 % and 1.58–20.54 %, respectively in the rhizoengineered tomato plants' leaves (Fig. 1D and E). Relative water content (RWC) is the major index of physiological water status of the plants. The maximum RWC (68.02 %) was noted in ESM14-treated plants' leaves which was not significantly different with ESK17, ESM17 and ESM24 (Fig. 1D). However, RWC was statistically similar with ESM4, ESM12 and in the control treatment. In this study, the highest proline accumulation (2.96 μg g^−1^ FW) was recorded in ESM12-treated plants' leaves, which was statistically analogous to ESK12, ESK17 and the control treatment (Fig. 1E). The minimum proline (1.71 μg g^−1^ FW) was detected in ESM4-treated plants’ leaves, followed by ESM17, ESM24 and ESM14 (Fig. 1E).

Catalase (CAT) is one of the most important plant protectant enzymes. CAT production was augmented 9.05–31.69 % by application of BPPGPR strains as compared to the control treatment (Fig. 1F). Ascorbate peroxidase (APX) plays a crucial role in controlling levels of ROS and operates within various subcellular compartments**.** In this study, APX production in all bacterial-treated plant leaves was significantly reduced by 38.77–85.55 % as compared to the control treatment (Fig. 1G). The highest APX production was detected to be 1.38 mmol min^−1^ mg^−1^ protein in the control treatment and the lowest APX (0.200 mmol min^−1^ mg^−1^ protein) was found by application of ESK12.

### 3.3 Rhizoengineering with BPPGPR-strains modulate photosynthetic pigments and gas exchange features

**Fig. 2:**
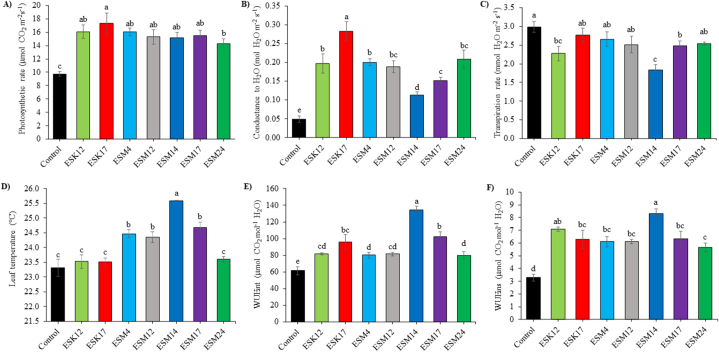
Effect of biofilm producing PGPR strains on gas exchange parameters of treated and untreated control plants. Photosynthetic rate (A), conductance to H_2_O (B), transpiration rate (C), leaf temperature (D), intrinsic water use efficiency (WUE_int_) (E) and instantaneous water use efficiency (WUE_ins_) (F). The distinct letters above the vertical bars signify statistically significant differences among treatments at a significance level of 5 % (P < 0.05), as determined by Fisher's LSD test. The error bars are the standard error obtained from three replicated treatments.

**Supplementary Fig. 1:**
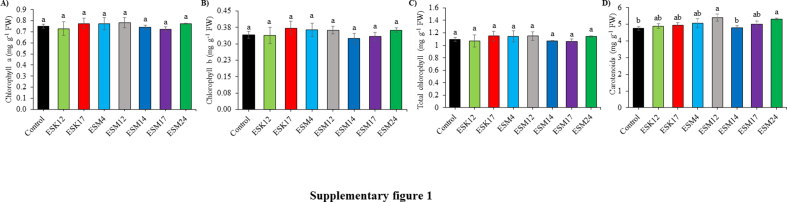
Effect of BPPGPR strains on leaf pigment content of tomato plant: chlorophyll *a* (A), chlorophyll *b* (B), total chlorophyll content (C) and carotenoid (D). The distinct letters above the vertical bars signify statistically significant differences among treatments at a significance level of 5 % (P < 0.05), as determined by Fisher’s LSD test. Additionally, the error bars are the standard error obtained from three replicated treatments.

The photosynthetic pigment content of tomato leaves for Chl *a*, Chl *b*, total chlorophyll and carotenoid were determined in both treated and non-treated control plants (Supplementary Figs. 1A–D). Chl *a*, Chl *b* and total chlorophyll content in all of the tested plants' leaves were statistically similar. However, significant changes were observed in carotenoid content. ESM12 and ESM24 produced 13.58 and 11.79 % more carotenoids than the non-treated control plants (Supplementary Fig. 1D). Photosynthetic attributes of tomato plants were assessed by measuring Pn, g_s_, E, LT, WUE_int_ and WUE_ins_ of leaves (Fig. 2A–F). Rhizoengineering with BPPGPR strains significantly increased Pn, g_s_, LT, WUE_int_ and WUE_ins_ by 47.34–78.07 %, 125.26–463.46 %, 0.91–9.78 %, 30.04–118.02 % and 71.86–152.45 %, respectively (Fig. 2A, B, D-F). On the contrary, E was reduced by 7.08–38.33 % in treated plants’ leaves (Fig. 2C). Maximum Pn and g_s_ was observed in plants inoculated with ESK17 (17.31 μmol CO_2_ m^−2^s^−1^ and 0.28 mol H_2_O m^−2^ s^−1^, respectively). ESM14-treated plants produced the highest level of LT, WUE_int_ and WUE_ins_ (25.59 °C, 134.26 μmol CO_2_ mol^−1^ H_2_O and 8.29 μmol CO_2_ mol^−1^ H_2_O, respectively) which also showed the lowest E value (1.84 mmol H_2_O m^−2^ s^−1^) (Fig. 2A–F).

### 3.4 Rhizoengineering with BPPGPR-strains increased yield, antioxidant and mineral contents in tomato

**Fig. 3:**
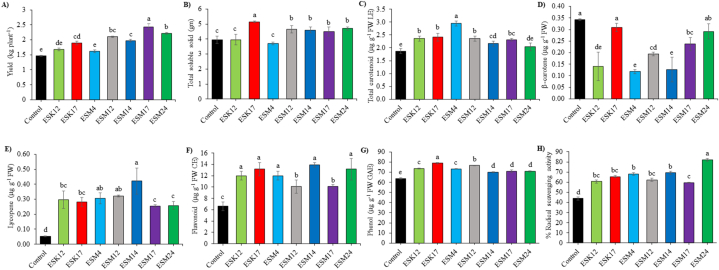
Impact of BPPGPR strains on yield (A), total soluble solid (B), total carotenoids (C), β- Carotene (D), lycopene (E), total flavonoids (F), total phenolic compounds (G), and total antioxidant activity (H). The distinct letters above the vertical bars signify statistically significant differences among treatments at a significance level of 5 % (P < 0.05), as determined by Fisher's LSD test. Additionally, the error bars are the standard error obtained from three replicated treatments.

All of the BPPGPR-inoculated plants except for ESM4 produced significantly more yield compared to the control plants. The highest yield was observed by application of ESM17 (2.435 kg plant^−1^) followed by ESM24 (2.215 kg plant^−1^). Percent increase in yield compared to the control treatment varied from 10.24 to 66.21 % (Fig. 3A). Total soluble solid (TSS), total carotenoids, lycopene, β-carotene, total flavonoids, total phenolic content and total antioxidant expressed as % radical scavenging activity in tomato fruits were also determined in both BPPGPR-treated and non-treated tomato fruits. A slight increase in TSS and total carotenoids was observed in the treated tomato fruits (Fig. 3B and C). The maximum TSS was attained by application of ESK17, which was found significantly different from all other treatments including control. The lowest TSS was found in ESM4 which was statistically similar with ESK12 and the control treatment (Fig. 3B). Unexpectedly, β-carotene content in treated tomato fruits was decreased (Fig. 3D), while lycopene content was increased significantly in all the treated plants, ranging from 4.8 to 7.94 times than the non-treated fruits (Fig. 3E). A significant increase in total flavonoids (52.32–110.46 %), phenolic contents (9.79–23.50 %) and total antioxidant activity (34.09–86.36 %) was detected in all bacterial-treated tomatoes as compared to the control treatment (Fig. 3F–H).

Mineral acquisitions were determined in both BPPGPR-treated and non-treated ripened tomatoes. Ca, Mg, Fe, Cu and Zn concentration in ripe tomatoes were found significantly higher in all BPPGPR-treated samples (Table 2). K content in all other samples except for ESM12, reflected significantly higher values than control. However, Na content was significantly lower in all of the treated samples as compared to the control treatment (Table 2). So, a considerable amount of changes in the fruit quality has been observed in the fruits of BPPGPR-treated tomato plants.

**Table 2:** Mineral contents in ripe tomato fruits as influenced by BPPGPR.

Strains	Mineral content in tomato (mg kg^−1^)							Bacterial colony forming unit (CFU) gm^−1^ rhizosphere (soil and root)
	Na	K	Ca	Mg	Fe	Cu	Zn	
Control	180.15 ± 1.34 a	2800.35 ± 9.18 e	80.40 ± 2.68 d	200.70 ± 3.39 f	11.34 ± 0.70 d	2.74 ± 0.05 f	4.70 ± 0.06 h	3.24E+5 ± 4.85E+4 c
ESK12	168.35 ± 1.26 bc	2910.15 ± 9.04 c	93.45 ± 3.88 c	222.35 ± 3.88 e	23.12 ± 1.05 a	3.08 ± 0.04 e	8.92 ± 0.06 c	4.71E+10 ± 3.12E+8 b
ESK17	163.60 ± 2.68 c	2857.25 ± 8.61 d	89.20 ± 4.38 cd	240.00 ± 2.96 d	17.15 ± 0.90 b	3.37 ± 0.06 c	10.31 ± 0.07 b	4.52E+10 ± 6.37E+8 b
ESM4	154.10 ± 1.27 d	2999.95 ± 9.74 a	112.65 ± 4.59 b	260.95 ± 3.04 c	14.10 ± 0.78 cd	3.19 ± 0.04 de	6.53 ± 0.03 f	5.86E+10 ± 4.24E+8 a
ESM12	169.51 ± 1.97 b	2786.35 ± 8.83 e	140.10 ± 5.51 a	300.05 ± 2.75 a	23.11 ± 1.04 a	3.32 ± 0.04 cd	12.98 ± 0.04 a	4.12E+10 ± 6.54E+9 b
ESM14	110.15 ± 1.20 f	2994.55 ± 8.33 a	121.40 ± 4.52 b	279.2 ± 1.55 b	18.67 ± 1.17 b	3.65 ± 0.06 b	6.735 ± 0.04 e	4.29E+10 ± 7.07E+8 b
ESM17	150.45 ± 1.76 d	3012.50 ± 10.10 a	150.00 ± 2.82 a	244.65 ± 3.60 d	17.35 ± 0.80 b	4.55 ± 0.05 a	7.80 ± 0.03 d	4.37E+10 ± 2.45E+8 b
ESM24	136.40 ± 1.69 e	2966.00 ± 9.81 b	109.85 ± 5.02 b	264.70 ± 3.56 c	16.40 ± 1.25 bc	3.15 ± 0.02 e	6.36 ± 0.05 g	4.33E+10 ± 3.24E+8 b

The data presented are means ± standard deviations of three replicated treatments. Different letters in columns indicate significant difference among treatments at 5 % (P < 0.05) level obtained from LSD test.

Moreover, serial dilution method was applied to determine the total bacterial colonies in the soil and roots collected from tomato root zone of all treatments under this study during harvesting of the fruits. A significant increase in bacterial colony forming units (CFU) was observed in per g of root and soil mixture in all BPPGPR-treated plots compared to the control plots (Table 2).

### 3.5 Principal component analysis (PCA) and clustered heat map

**Fig. 4:**
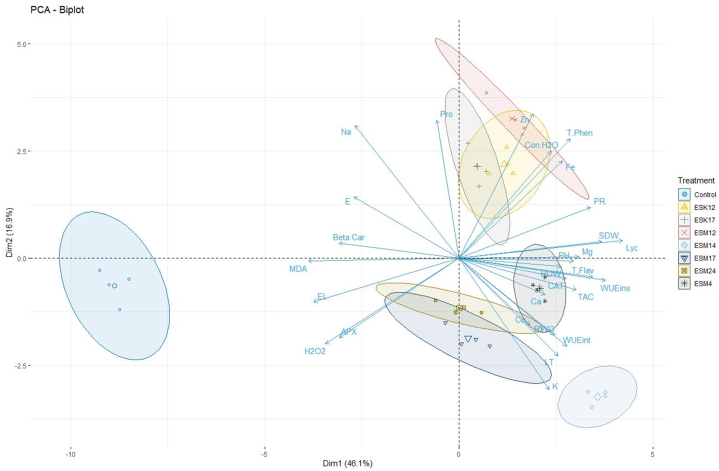
Principal component analysis (PCA) to elucidate treatment-variable interactions in both PGPR treated and non-treated tomato plants under field conditions. The arrows originating from the central point of biplots represent negative or positive correlations of different variables, whereas their proximity exposes correlation strength with specific treatment. The included variables are PH (plant height), RDW (root dry weight), SDW (shoot dry weight), Yield, Pro (proline), EL (electrolyte leakage), MDA (malonaldehyde content), RWC (relative water content), CAT (catalase), APX (ascorbate peroxidase), H2O2 (hydrogen peroxide), Beta-Car (β- Carotene), Lyc (lycopene), T Flav (total flavonoid), T Phen (total phenol), TAC (total antioxidant content/% radical scavenging activity), PR (photosynthetic rate), Con H2O (stomatal conductance to H_2_O), E (transpiration rate), LT (leaf temperature), WUEint (intrinsic water use efficiency), WUEins (instantaneous water use efficiency), Na (sodium), K (potassium), Ca (calcium), Mg (magnesium), Fe (iron), Cu (copper) and Zn (zinc).

A PCA biplot in Fig. 4 represents the 2D estimate of the original multidimensional space for our experimental dataset where each ellipsoid represents the treatments (bacterial strains including control) and the axes represent the variables. Each variable was represented as vector in the biplot and their relative length indicates the proportion of variability. The closeness of points indicates that the corresponding treatments have similar proﬁles in this biplot and the points further away as unrelated proﬁles. From the biplot, it can be easily observed that the treatment-control is far away from all other BPPGPR treatments indicating the dissimilarity profiles. The approximate value of any point on the plot can be explained based on the axes. Fig. 4 shows that 46.1 % and 16.9 % of the variation in the original variables for plant height, root and shoot dry weight, proline, H_2_O_2_, electrolyte leakage, MDA, relative water content, catalase, APX, photosynthetic rate, leaf temperature, conductance to H_2_O, WUE_int_, WUE_ins_, transpiration rate, β-carotene, lycopene, total soluble solid, total phenolics, total flavonoids, radical scavenging activity, and fruit minerals (Na, K, Ca, Mg, Fe, Zn and Cu) are explained by principal component 1 (PC1) and 2 (PC2) in the multivariate space. In the case of PC1, catalase and H_2_O_2_ reside on opposite directions, which infers that treatments exhibiting a higher degree of catalase may exhibit lower H_2_O_2_ values and vice-versa, which was also found in the biochemical analysis results (Fig. 1A–F). Moreover, the higher the score of a dot (treatment) towards the direction of a PC, the stronger it correlates to that PC and vice-versa. So, the variables e.g., H_2_O_2_, MDA, EL, APX, Na, E, β-carotene contributed more towards control treatment (Fig. 4).

**Fig. 5:**
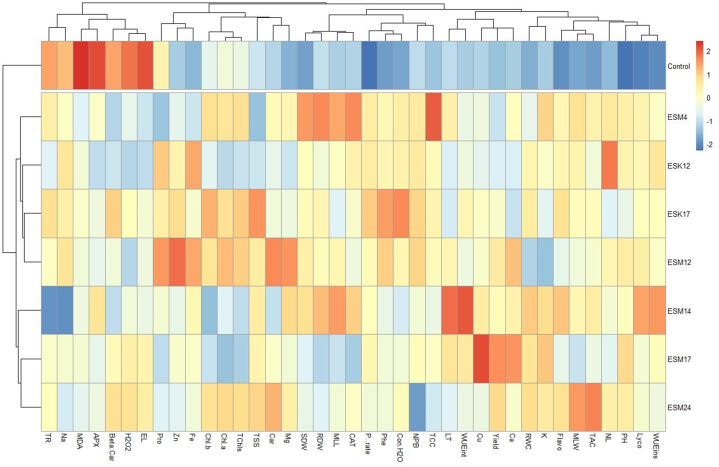
Clustered heatmap showing treatment-variable interactions in both PGPR treated and non-treated tomato plants under field conditions. The normalized mean values of different parameters were clustered. The color scale indicates the strength of the normalized mean values for different parameters. The variables included PH (plant height), NL (number of leaves), NPB (number of primary branch), MLL (maximum leaf length), MLW (maximum leaf width), RDW (root dry weight), SDW (shoot dry weight), Chl a (Chlorophyll *a*), Chl b (Chlorophyll *b*), TChls (Total chlorophyll), Car (carotenoids), Yield, Pro (proline), EL (electrolyte leakage), MDA (malonaldehyde content), RWC (relative water content), CAT (catalase), APX (ascorbate peroxidase), H2O2 (hydrogen peroxide), Beta-Car (β- Carotene), Lyco (lycopene), Flavo (total flavonoid), Phe (total phenol), TAC (total antioxidant content/% radical scavenging activity), TSS (total soluble solids), TCC (total carotenoid content), P. rate (photosynthetic rate), Con H2O (stomatal conductance to H_2_O), TR (transpiration rate), LT (leaf temperature), WUEint (intrinsic water use efficiency), WUEins (instantaneous water use efficiency), Na (sodium), K (potassium), Ca (calcium), Mg (magnesium), Fe (iron), Cu (copper) and Zn (zinc). (For interpretation of the references to color in this figure legend, the reader is referred to the Web version of this article.)

Based on the data obtained from different variables, a clustered heat map was also developed which clearly demonstrated two separate clusters of studied treatments. Non-treated plants grouped in one cluster and the rest of the PGPR-treated plants formed another cluster (Fig. 5). In the case of variables, stress-related parameters which negatively influenced plant growth (EL, H_2_O_2_, MDA, APX, β-carotene, TR, Na) formed a distinct cluster group (Fig. 5). It is clearly visible in the heat map that the PGPR-treated plants recovered from these stress conditions by showing lower values compared to the non-treated plants. Moreover, growth-related parameters, leaf pigments, fruit quality attributes, antioxidants and gas exchange features came under another cluster and further sub-clustered based on their normalized data values and correlation (Fig. 5). A completely opposite trend was found in non-stress clustering groups where the PGPR-treated plants showed higher mean values compared to the non-treated plants (Fig. 5).

## 4 Discussion

The use of PGPR strains is considered as an environment-friendly approach for tomato production [77]. Fenta and Assefa [78] reported that *Pseudomonas* isolates significantly increased root, shoot length and biomass in tomato under greenhouse condition. Similar results were also found in tomato by inoculation of *Pseudomonas* APF1, *B. subtilis* B2G, and rhizobacterial isolates *B. cereus* BC1AW and *P. putida* PP3WT [79,80]. According to Patten and Glick [81], PGPR strains are reported to produce different phytohormones related to promotion of plant growth. Indole-3-acetic acid (IAA) is a phytohormone, produced by the PGPR strains. IAA is responsible for increasing lateral roots and the number of root hairs which eventually can enhance nutrient and water uptake in plants. Ethylene is an another phytohormone, when produced in higher amounts is reported to reduce root and shoot development in plants [82]. PGPR also produce 1-aminocyclopropane-1-carboxylate (ACC) deaminase that breaks the precursor of ethylene, ultimately diminishing the amount of ethylene in plants [33]. PGPR strains were also shown to solubilize minerals like P, K, Zn and Fe and fix atmospheric N, leading to stimulate growth of the plants [83]. It is often reported that PGPR-mediated biofertilizers perform better only in the controlled glasshouse or laboratory conditions but remains unsuccessful to produce expected result under field conditions [32]. In this study, biofilm producing plant growth promoting rhizobacteria (BPPGPR) were inoculated (termed rhizoengineering) in order to solve this problem as recommended by Ahkami et al. [27]. All the rhizoengineered plants increased growth under N-deficient field conditions (Table 1). These BPPGPR strains previously shown to be solubilized P, fixed atmospheric N, produced IAA, ACC deaminase, siderophores, ammonia and biofilm [64]. The EPS produced by these biofilm producing bacterial strains was also previously characterized by Fourier-transform infrared spectroscopy (FTIR), revealing polysaccharides, nucleic acids, lipids, and protein compounds were dominant [64]. Along with the protection of bacterial cells against any stress, EPS can also provide carbon source to the adjacent microbial community [84,85]. As a result, the colony forming units in the rhizospheric zone are found to be higher (Table 2) and may have solubilized nutrients at a faster rate for higher plant uptake and increased growth. In this study, rhizoengineered plants exhibited increased photosynthetic features such as photosynthetic rate, conductance to H_2_O, leaf temperature and both intrinsic and instantaneous water use efficiency (Fig. 2A,B,D-F and 5). Transpiration rate was found to be lower in rhizoengineered plants (Fig. 2C). Thus, the increasing plant growth under N-deficient field conditions could be the result of solubilization of minerals, fixation of atmospheric N, production of IAA and ACC deaminase, and induction of photosynthetic features as reported [64].

Nutrient deficiency is one of the abiotic stress conditions for plants, and a major constraint against crop production [86,87]. Nutrient starvation can result in higher ROS production in plants due to oxidative stress [14,[88], [89], [90]]. H_2_O_2_ is an indicator of ROS [91]. Based on soil analysis, our experimental field experiment was nutrient deficient i.e., low fertile [65] to impose nutrient deficiency stress on tomato plants. In this study, all the control tomato plants produced significantly higher H_2_O_2_ (Fig. 1, Fig. 5). Electrolyte leakage serves as an indicator of the extent of damage to the cell membranes in plant cells. Greater cell integrity and stability will result in more resistance against any stress conditions [92]. In this study, significantly higher electrolyte leakage is observed in plants without any treatment of bacterial strains (Fig. 1B), suggesting increased cell membrane damage and less resistance. Similar trend is also observed in the case of MDA (Fig. 1, Fig. 5) which is a result of lipid peroxidation in cells and also an oxidative stress indicator [93]. Leaf RWC of plants is used as an index of physiological water level [94]. Lower leaf RWC is related with closing of stomata which ultimately leads to reduced CO_2_ assimilation [95]. Except for control plants, all other rhizoengineered plants showed higher levels of RWC in leaf tissues (Fig. 1, Fig. 5). PCA data also indicated that untreated plants exhibited a higher level of positive correlation with stress-related attributes such as MDA, H_2_O_2_, EL, E, and APX than PGPR-treated plants (Fig. 4) indicating the alleviation of stress by application of BPPGPR. A similar trend was also visible in the clustered heat map (Fig. 5) revealing the confirmation of BPPGPR-treated plants recovered from stress conditions.

Application of PGPR could be an effective strategy for tackling both abiotic and biotic stresses which is mainly through the production of antioxidants in inoculated plants [96,97]. Antioxidant includes a number of non-enzymatic molecules and enzymes, which have the ability to scavenge ROS without undergoing any changes during the process [98]. Ascorbic acid, flavonoids, phenolics, glutathiones, α-tocopherol, chlorophyll derivatives, alkaloids, amino acids and amines, phytosiderophores, high (ferritin) and low (carotenoids, nicotianamine) molecular mass antioxidants are the non-enzymatic antioxidant compounds [[98], [99], [100]]. Primary antioxidant enzymes include APX, CAT, SOD, POD, GR, monodehydroascorbate reductase (MDHAR) and dehydroascorbate reductase (DHAR) [98,101]. Proline serves as an osmoprotectant and a stress marker which detoxifies stressed cells from the ROS [102]. Here, proline content in ESM12-engineered plant is found to be the highest which is statistically similar with ESK12, ESK17 and non-inoculated control plants (Fig. 1E), suggesting that these plants could be more stressed than the other rhizoengineered plants. In the case of, APX and CAT, both produced opposite results. Interestingly APX content is highest in the control treatment whereas CAT is lowest in the control treatment (Fig. 1F and G). In lettuce plants, application of *P. mendocina* and *Glomus intraradices* remarkably enhanced CAT under drought conditions [103]. In the present study, APX content in the rhizoengineered plants were found to be lower which differs from other available literatures. Heidari et al. [104] reported, application of *Pseudomonas* sp., *B. lentus* and *Azospirillum brasilens* significantly increased the APX content in basil. However, enhancement of antioxidant compounds in tomatoes by application of BPPGPR was not reported under field conditions.

Several PGPR strains were reported to increase mineral content in banana [105], strawberry [106], cucumber [107] and tomato [108]. Tomato yield, TSS and lycopene content were shown to be increased by application of *Bacillus subtilis*, *B. licheniformis*, *B. amyloliquefaciens*, *Priestia megaterium*, and *Kosakonia Radicincitans* under pot/field conditions [[61], [62], [63]]. Biofilm producing plant growth-promotion rhizobacteria (BPPGPR) including the genera *Pseudomonas, Bacillus* and *Enterobacter* isolated from tomato rhizosphere were reported to increase yield and bioactive contents in pot-grown tomatoes in addition to plant growth [54]. In pot-grown tomatoes by application of *P. poae* ESN2, *B. aryabhattai* ESN3, *B. megaterium* ESN4, *E. cloacae* ESN16, *P. monteilii* ESN26, *P. putida* ESN32, *P. fluorescens* ESN35, *P. extremorientalis* ESN36, and *P. chlororaphis* ESN37, phenolics, flavonoids and antioxidant capacity were also demonstrated to increase by 6.3–52 %, 10.4–69.2 % and 13.3–66.6 % over controls [57]. Bioactive compounds provide better human health benefits [109]. Lycopene offers numerous human health benefits such as maintaining good heart health, reducing cardiovascular diseases and prostate cancer as well as scavenging the singlet and reactive oxygen groups [110,111]. In this study, rhizoengineering with BPPGPR strains significantly increased the growth, yield, TSS, minerals, total carotenoids, lycopene, total flavonoids, phenolic compounds and total antioxidant activity compared to the control tomato plants under N-deficient field conditions. So far, increments of yield and bioactive compounds by rhizoengineering with BPPGPR strains under N-deficient field conditions were not reported by any other researchers.

## 5 Conclusions

In conclusion, the application of BPPGPR strains through rhizosphere manipulation had a positive impact on the growth, physiology, and yield of tomato plants. It improved plant growth parameters, photosynthetic pigments, stress tolerance, gas exchange features, fruit quality and mineral content. These results suggest that applied strains can be utilized as a potential biotechnological tool to enhance tomato crop productivity, quality, and stress tolerance, contributing to sustainable agriculture and food security. To further optimize the practical application of BPPGPRs, future research should focus on several key areas such as conducting mechanistic studies to elucidate valuable insights into their mode of action in plants, field trials under diverse environmental conditions and soil types to help validate their efficacy in real-world agricultural settings and exploring a wider diversity of BPPGPR strains from various ecological niches to uncover novel strains with unique attributes. Additionally, investigating the long-term impact of BPPGPR strains on soil health and the overall plant microbiome will be crucial for sustainable agricultural practices. Finally, comparative studies with conventional growth-promoting agents will provide a comprehensive understanding of the relative effectiveness and practicality of PGPR strains in modern agriculture.

## Data availability

Data will be made available on request.

## CRediT authorship contribution statement

**Md. Manjurul Haque:** Writing – review & editing, Supervision, Resources, Project administration, Methodology, Investigation, Funding acquisition, Conceptualization. **Md. Rahat Bari Rupok:** Visualization, Formal analysis, Data curation. **Abul Hossain Molla:** Writing – review & editing, Supervision. **Md. Mizanur Rahman:** Supervision, Investigation. **Habibul Bari Shozib:** Supervision, Investigation, Formal analysis. **Md Khaled Mosharaf:** Writing – original draft, Validation, Supervision, Methodology, Investigation, Formal analysis, Data curation, Conceptualization.

## Declaration of competing interest

The authors declare that they have no known competing financial interests or personal relationships that could have appeared to influence the work reported in this paper.
