# Supplementary material for: Rhizoengineering with biofilm producing rhizobacteria ameliorates oxidative stress and enhances bioactive compounds in tomato under nitrogen-deficient field conditions
Source: Heliyon. 2024 Jul 8;10(14):e34276. doi: 10.1016/j.heliyon.2024.e34276 (PMC11301190; doi:10.1016/j.heliyon.2024.e34276)
Supplement: Multimedia component 1 [file mmc1.docx]

| **Parameters** | **df** | **F value** | **Pr(>F) value** |
| --- | --- | --- | --- |
| Plant height | 7 | 5.175 | 0.00307** |
| Number of leaves | 7 | 8.024 | 3e-04*** |
| Maximum leaf length | 7 | 4.884 | 0.00414** |
| Maximum leaf width | 7 | 3.294 | 0.0229* |
| Root dry weight | 7 | 7.32 | 0.000425*** |
| Shoot dry weight | 7 | 83.27 | 2.17e-11*** |
| Hydrogen peroxide | 7 | 84.85 | 1.88e-11*** |
| Electrolyte leakage | 7 | 5.223 | 0.00299 ** |
| Malondialdehyde | 7 | 5.707 | 0.00191 ** |
| Relative water content | 7 | 10.11 | 7.58e-05 *** |
| Proline | 7 | 5.018 | 0.00363 ** |
| Catalase | 7 | 14.11 | 9.12e-06 *** |
| Ascorbate peroxidase | 7 | 65.96 | 1.3e-10 *** |
| Chlorophyll a | 7 | 0.355 | 0.915^ns^ |
| Chlorophyll b | 7 | 0.488 | 0.829 ^ns^ |
| Total chlorophyll | 7 | 0.408 | 0.884 ^ns^ |
| Carotenoid | 7 | 1.901 | 0.136 ^ns^ |
| Photosynthetic rate | 7 | 6.106 | 0.00135 ** |
| Conductance to H_2_O | 7 | 29.96 | 4.81e-08 *** |
| Transpiration rate | 7 | 4.249 | 0.00788 ** |
| Leaf temperature | 7 | 19.28 | 1.1e-06 *** |
| Intrinsic water use efficiency | 7 | 19.03 | 1.21e-06 *** |
| Instantaneous water use efficiency | 7 | 11.12 | 4.22e-05 *** |
| Yield | 7 | 20.07 | 8.36e-07 *** |
| Total soluble solid | 7 | 12.99 | 1.57e-05 *** |
| Total carotenoid | 7 | 25.25 | 1.65e-07 *** |
| β-Carotene | 7 | 20.56 | 7.07e-07 *** |
| Lycopene | 7 | 33.15 | 2.3e-08 *** |
| Phenol | 7 | 156.1 | 1.62e-13 *** |
| Flavonoid | 7 | 16.05 | 3.86e-06 *** |
| Total Antioxidant | 7 | 12.64 | 1.87e-05 *** |
| Na | 7 | 168.4 | 8.93e-14 *** |
| K | 7 | 98.52 | 5.9e-12 *** |
| Ca | 7 | 32.68 | 2.55e-08 *** |
| Mg | 7 | 99.68 | 5.39e-12 *** |
| Fe | 7 | 17.07 | 2.55e-06 *** |
| Cu | 7 | 116.9 | 1.55e-12 *** |
| Zn | 7 | 2610 | <2e-16 *** |
| Bacterial colony | 7 | 53.24 | 6.67e-10 *** |

**Supplementary table 1**. F and P values obtained from ANOVA for different parameters in the study. All the parameters were found significant at least at 5 % (P < 0.05) significance level except chlorophyll a, b, total chlorophyll, and carotenoid.

(***) indicate significant difference at 0.1 % (p ≤ 0.001) level of significance

(**) indicate significant difference at 1 % (p ≤ 0.01) level of significance

(*) indicate significant difference at 5 % (p ≤ 0.05) level of significance

(^ns^) indicate no significant difference
